# Beyond Condoms: Risk Reduction Strategies Among Gay, Bisexual, and Other Men Who Have Sex With Men Receiving Rapid HIV Testing in Montreal, Canada

**DOI:** 10.1007/s10461-016-1344-7

**Published:** 2016-03-09

**Authors:** Joanne Otis, Amélie McFadyen, Thomas Haig, Martin Blais, Joseph Cox, Bluma Brenner, Robert Rousseau, Gilbert Émond, Michel Roger, Mark Wainberg, The Spot Study Group

**Affiliations:** 1 Department of Sexology, Université du Québec à Montréal, Case postale 8888, succursale Centre-ville, Montreal, QC H3C 3P8 Canada; 2 CIHR Canadian HIV Trials Network, Vancouver, Canada; 3 COCQ-SIDA, Montreal, QC Canada; 4 Direction de santé publique du CIUSSS du Centre-Sud-de-l’Île-de-Montréal, Montreal, QC Canada; 5 Faculty of Medicine, McGill University, Montreal, QC Canada; 6 Applied Human Sciences, Concordia University, Montreal, QC Canada; 7 Laboratoire de Biologie Moléculaire, Centre hospitalier de l’université de Montréal, Montreal, QC Canada; 8 RÉZO, Montreal, QC Canada

**Keywords:** Risk-reduction strategies, Condom use, Men who have sex with men, Latent class analysis, Combination HIV prevention

## Abstract

Gay, bisexual, and other men who have sex with men (MSM) have adapted their sexual practices over the course of the HIV/AIDS epidemic based on available data and knowledge about HIV. This study sought to identify and compare patterns in condom use among gay, bisexual, and other MSM who were tested for HIV at a community-based testing site in Montreal, Canada. Results showed that while study participants use condoms to a certain extent with HIV-positive partners and partners of unknown HIV status, they also make use of various other strategies such as adjusting to a partner’s presumed or known HIV status and viral load, avoiding certain types of partners, taking PEP, and getting tested for HIV. These findings suggest that MSM who use condoms less systematically are not necessarily taking fewer precautions but may instead be combining or replacing condom use with other approaches to risk reduction.

## Resumen

Los hombres gay bisexuales y otros hombres que tienen sexo con hombres (HSH) han adaptado sus prácticas sexuales en el transcurso de la epidemia de VIH/sida de acuerdo con los datos y el conocimiento disponibles en materia de VIH. Este estudio trata de identificar y comparar los patrones de uso del condón entre homosexuales, bisexuales y otros HSH que se hicieron la prueba de detección de VIH en un sitio comunitario en Montreal, Canadá. Los resultados mostraron que mientras los participantes del estudio usan condones en cierta medida con parejas VIH-positivas y con aquellas que desconocen su estado de VIH, también recurren a varias otras estrategias tales como seleccionar sus parejas de acuerdo al estado de VIH (supuesto o conocido) o de acuerdo a la carga viral, evitar ciertos tipos de parejas, recurrir a la PEP, y hacerse las pruebas de detección de VIH. Estos hallazgos sugieren que los HSH que usan condones de una manera menos sistemática no están tomando necesariamente menos precauciones si no que en cambio pueden estar combinando o reemplazando el uso del condón con otros enfoques de reducción de riesgos.

## Introduction

Gay, bisexual, and other men who have sex with men (MSM) remain over-represented in global data for the HIV epidemic [1–3]. Rates of HIV infection in this population continue to rise despite ongoing prevention efforts and new prevention tools. In Quebec, MSM accounted for 61 % of new diagnoses in 2014 and incidence among MSM under 35 years of age has climbed since 2003, suggesting that sexual risk behaviors such as condomless anal sex may be increasing over time [4]. However, a decrease in condom use could be due to greater use of other risk reduction strategies that at present are insufficiently documented and understood [5–9].

### Combination Prevention and the Ongoing Need for Behavioral Approaches

Understanding the extent to which MSM are using a diversified range of risk reduction strategies has become increasingly important. This diversification includes new biomedical prevention options such as PrEP but also extends to the prevention impact of antiretroviral therapy used to treat HIV infection. Yet even as these biomedical strategies show promise, behavioral strategies continue to play a critical role. In some modeling of the epidemic among MSM, for example, researchers have found that the prevention benefits of antiretroviral therapy erode unless it is implemented sufficiently at the community level and accompanied by adequate condom use and testing [1–4, 10, 11].

These models point to the importance of combination prevention, the concurrent and strategic use of a range of biomedical, behavioral, and socio-structural interventions. Implemented at multiple levels (individual, community, societal), combination prevention strategies integrate various prevention options to be used before, during, or after sex to address the needs of specific populations and different modes of transmission [12]. If adequately implemented, these combined approaches could significantly reduce the number of new infections among MSM [5, 13–19]. Yet combination prevention presents challenges at the individual level with respect to using different behavioral strategies together (condoms, non-condom-based HIV risk reduction strategies) and combining these in turn with biomedical strategies such as pre-exposure prophylaxis (PrEP), post-exposure prophylaxis (PEP), and testing. Understanding how MSM are navigating these challenges as well as the ways in which they may be combining strategies or substituting one strategy for another are priorities for research and intervention development.

### Seroadaptive and Biomedical Risk Reduction Strategies: Combined with Condoms or Used as a Replacement?

In the early 1990s, the urgency of the response to HIV in gay communities began to fall off [20]. Since then, condom use during anal sex has declined over a number of years [21–24] and increases in condomless anal sex have been observed in gay communities around the world [25–32] including Montreal [33–35]. In parallel, there has been an increase in the use of behavioral risk-reduction strategies and in particular seroadaptive practices, a term that refers to various non condom-based approaches to sexual risk reduction that are based on knowledge of one’s own HIV status and the status of sexual partners [5, 6, 36, 37].

These strategies include negotiated safety (an agreement that allows for condomless anal sex between partners who have the same HIV status, are in a relationship, and agree that any sex with partners outside the relationship must be protected) [38, 39], withdrawal (during condomless anal sex, the insertive partner withdraws before ejaculation) [39, 40], strategic positioning (the practice of having HIV-negative partners take the insertive position and HIV-positive partners the receptive position during condomless anal sex) [36, 39, 41, 42], serosorting (only having condomless anal sex with partners of the same HIV status) [37, 39, 42–46], and taking viral load into consideration (the use of viral load test results to assess whether condomless anal sex between serodiscordant partners poses a risk for HIV transmission) [22, 47]. In studies among MSM, 25–75 % of respondents reported using seroadaptive strategies [5–8, 42, 48], with 25–50 % saying they used serosorting [7, 8, 37, 45, 49, 50] and 6–30 % reporting the use of strategic positioning [5, 6, 8, 32, 48]. Consideration of viral load is less documented and only a small proportion of MSM appear to use it for risk reduction [32].

The risk of HIV transmission from condomless anal sex varies depending on the seroadaptive practice being considered as well as multiple contextual factors such as the prevalence of acute infections in the community [8, 39, 44, 46, 51–55]. Overall, these practices are somewhat effective compared to using no other strategy for condomless anal sex, but less effective than consistent condom use or not having condomless anal sex [39, 42]. With respect to viral load, researchers were able to show as early as 2000 that there is no risk of transmission if HIV viral load is below 1500 copies/ml [55]. The results of an ongoing study of serodiscordant MSM couples have been promising, with no HIV transmission observed over 2 years in couples where the HIV-positive partner has an undetectable viral load [56].

The risk level for HIV infection through anal sex also varies depending on the use of available biomedical strategies such as testing, PEP, and PrEP. Testing is crucial to combination prevention [57–60] since the effectiveness of seroadaptive strategies depends on individuals having accurate knowledge about their own HIV status and the status of their sexual partners. Yet recent studies have found that 8–20 % of MSM had never been tested for HIV and only 60 % had been tested in the past 12 months [32, 61, 62]. Post-exposure prophylaxis [PEP], the provision of antiretroviral drugs after a possible exposure to HIV [63, 64], can stop an HIV infection from taking hold if taken within 1–3 days of exposure [59, 63, 65–67]. In a range of studies among MSM, 36–48 % of participants said they were aware of PEP [68–70] but only 1.9–6.3 % had used it [32, 68, 69, 71, 72] reflecting a broader trend in which use of PEP by MSM has remained well below 10 %. Pre-exposure prophylaxis [PrEP], the use of antiretroviral drugs by an HIV-negative person on either a daily or intermittent basis prior to sex, has been shown to reduce the risk of contracting HIV in a number of clinical trials among MSM and transgender participants [73–75]. Until recently, researchers have found awareness of PrEP among MSM to be low [69, 70] with only a small minority saying they had used it [69], but PrEP is now among the risk reduction strategies that an increasing number of MSM may be using.

If consistent condom use during anal sex remains important for many MSM [5, 7], seroadaptive and biomedical strategies have not necessarily been perceived or constructed as something to be used in combination with condoms and in some instances may be viewed as substitutes for condom use that help to reconcile risk reduction with pleasure [9]. In the current paradigm shift towards combination prevention, the dichotomy between condom use and other strategies will create a range of challenges for individuals and communities as well as new challenges in terms of public health.

### Objectives of the Study

This study analyzes the risk reduction strategies (condom-based, non-condom-based, and biomedical) reported by MSM who received rapid HIV testing at “Spot”, a community-based testing site in Montreal. A latent class analysis [LCA] [76] was performed based on several indicators of condom use in relation to anal sex practices (e.g. position as top or bottom) and the HIV status of sexual partners, allowing us to identify sub-groups of participants who responded in similar ways with respect to these indicators. Participants were assigned to latent classes based on probability of class membership for specific patterns of condom use.

LCA was chosen because it is a type of analysis that makes it possible to observe distinct subgroups, providing information that can help to improve and adapt interventions. LCA has been used in a number of recent studies to examine patterns of substance use [77, 78], online and offline sexual health-seeking [79], and syndemic factors related to HIV infection [80] among MSM. To our knowledge, this strategy has not been previously used to segment a participant sample in order to analyze the patterns of combination and substitution that characterize how MSM use condom-based, non-condom-based, and biomedical risk reduction strategies.

The larger goals of this analysis were to identify patterns in the use of behavioral and biomedical strategies and understand whether study participants who use condoms inconsistently or not at all have actually stopped taking precautions or if they have simply replaced or combined condom use with other risk reduction strategies. The diversification of risk reduction strategies is likely to continue and better understanding of these issues will allow for better targeting and tailoring of interventions aimed at MSM from a combination prevention perspective.

## Methodology

### Study Design and Data Collection

Since 2009, the Spot project has offered free, anonymous, rapid HIV testing for men who have sex with men at a community-based testing site in close proximity to Montreal’s gay village. Participants were recruited between July 2009 and July 2012 using the following inclusion criteria: self-identification as male; 18 years of age or older; ability to speak and understand French or English; resident of Quebec; anal sex with another man in the past 12 months; and unknown HIV status at the time of testing. Individuals with symptoms of possible HIV infection were excluded from the study and referred to a clinic.

Recruitment was supported through outreach activities organized by community workers from RÉZO (a community organization) in a range of community and social venues. Over a quarter (26.6 %) of participants said they were referred to Spot by friends, with smaller proportions saying they heard about the project by means of print materials such as flyers or cards (11.1 %); articles (9.8 %) or print advertisements (8.7 %) published in community magazines; search engines (7.3 %); or online advertising (3.3 %).

Participants could book an appointment by phone or in person. Prior to testing, a baseline questionnaire was administered by a community worker or nurse that took approximately 30 min to complete. Participants were then tested for HIV using the *INSTI™* rapid HIV test (bioLytical Laboratories) and received the test result (negative or reactive) a few minutes later. All reactive results were confirmed independently either by the *Laboratoire de santé publique du Québec* (venous blood draw analysis) or the McGill AIDS Centre (dry bloodspot analysis). Between July 2009 and July 2012, 1, 855 clients who met the inclusion criteria came to Spot for HIV testing and of these, 93.8 % accepted to participate in the study. Those who declined to take part were provided with the same testing and counseling services as study participants but not required to complete the questionnaire. No data were collected that would allow for comparison with participants. Time constraints were the main reason given for not wanting to participate in the study.

### Measures

#### Condom Use Indicators According to Type of Partner and Type of Sexual Activity

Twelve nominal condom-use indicators were used to perform the latent class analysis (LCA). These indicators were based on the following questions about condom use during anal sex in the past 3 months for two types of penetration (complete or partial) and two positions (insertive position as a “top” or receptive position as a “bottom”): “*Did you have anal sexual relations with male partners of unknown HIV status?”* If the answer was yes, participants were then asked: “*With these partners, did you do the following: a) put your penis all the way into their anus (complete penetration) (top)?; b) put your penis only part way into their anus (dipping) (top)? c) put their penis all the way into your anus? (complete penetration) (bottom)?; d) put their penis only part way into your anus (dipping) (bottom)?”* Participants who answered yes for any of these sexual activities were also asked: “*For what proportion of these relations did you use a condom?”* The choices were *none* (which was coded as “never uses a condom”); *the minority, half,* or *the majority* (all three of which were coded as “inconsistent condom use”); and *all* (which was coded as “systematic condom use”). The full set of questions as they appeared in the questionnaire can be found in Appendix 1. This pattern of questions was repeated for two other types of partners with whom there is a potential for HIV exposure: HIV-positive partners whose viral load is detectable or unknown; and HIV-positive partners whose viral load is undetectable.

#### Sociosexual and Health Profile

Participants were asked when they had last been tested for HIV (in the past 12 months/over 12 months ago) and whether they had ever used PEP. They were also asked to indicate how and where sexual partners were met in the past 3 months, including the proportion of sexual encounters that happened in saunas (less than half the time/half the time or more). Two items were used to assess participants’ attitudes toward HIV testing and HIV-related issues (“*Despite everything you hear about the criminalization of HIV transmission, you’d rather know your HIV status so you can make the right decisions for your own health and the health of others*.” and “*You feel you are at risk of being infected with HIV*.”). These items were measured using a scale ranging from 1 (“*Strongly disagree*”) to 7 (“*Strongly agree*”). Intentional condomless anal sex (“barebacking”) was measured by asking participants if they had intentionally had unprotected anal sex with a casual partner or during a one-night stand in the past 3 months (yes or no).

### Analysis Plan

We used LCA in the first instance to empirically identify patterns of condom use among Spot participants with respect to sexual partners with whom there could be a risk of exposure to HIV. The analysis was performed using Latent Gold 5.0 [76]. Given the semantic redundancy of the LCA indicators used to characterize the HIV status of sexual partners, we expected to observe residual correlations between pairs of indicators that were redundant and these residual pairs were correlated. Thus, we added direct effects between semantically redundant indicators. We performed the LCA for one-through eight-class models, without adding covariates, and we compared models based on BIC and AIC values and the interpretability of results. BIC and AIC are relative fit indices and as such, do not have cutoff values. Lower BIC and AIC values suggest better-fitting models [77–80]. While the AIC value pointed toward a six-class model, the BIC value suggested that a five-class model would be the best fit. For the purposes of interpretability, all classes needed to be clearly distinguishable from one another. This was not the case for the six-class model since it contained two classes that were nearly identical. Therefore, for the sake of interpretability and parsimony, we gave more weight to the BIC value and retained the five-class model. This model is described by two sets of parameters: estimates of class prevalence and probability that the members of a class endorse each condom-use indicator.

Each class of participants was then compared with the other classes to identify differences and similarities for participants’ sociosexual characteristics. Mean and distribution differences across classes for variables with respect to sociosexual profile were estimated using the omnibus Wald tests, the default option implemented in Latent Gold for such comparisons [76]. When the Wald’s statistics indicated statistical significance for group mean differences, post hoc pairwise comparisons between classes were performed. The familywise rate of Type I error for all pairwise comparisons was set at 0.05. Given that the pairwise comparisons for the five classes involved ten tests, the per-comparison Type I error rate was set at 0.005 (0.05/10). Because all of the variables used for univariate analysis (questions relating to risk reduction strategies or sexual behavior) were significant (*p* < 0.05) except for two, all were included for multivariate analysis. For this reason, we have only reported the results of multivariate analysis. Sociodemographic variables used to describe the sample included sexual orientation, age, education, and income. These variables were used as controls for multivariate analysis.

## Results

### Sample Description

Demographic information is presented in Table 1. The sample consists of 1740 individuals who were tested for HIV at Spot between July 2009 and July 2012. The average age of participants was 34 years (SD 10.4), 78.5 % spoke French as their first language, 80.5 % had a college diploma or higher, and 55.9 % had a personal annual income of at least $30,000. Most participants (82.8 %) self-identified as homosexual or gay, 12.6 % as bisexual, and 4.7 % as “other” (two-spirited, queer, heterosexual, or uncertain). With respect to place of birth, 37.8 % of participants reported being born outside Canada. Just over half (52.1 %) had been tested for HIV in the past 12 months, 11 % reported having used PEP at least once and 17.2 % said they had had intentional condomless anal sex in the past 3 months.

**Table 1:** Participants’ demographic and sociosexual characteristics (N = 1740)

	Mean (SD)	(95 % CI)
Age (years)	34.0 (10.4)	(33.3–43.3)

	N (%)	(95 % CI)
First language		
French	1366 (78.5)	(76.6–80.4)
English	374 (21.5)	(19.6–23.4)
Education		
High school diploma or less	303 (17.4)	(15.7–19.3)
College diploma or higher	1399 (80.5)	(78.5–82.2)
Other	36 (2.1)	(1.5–2.7)
Personal annual income		
Under 30,000$	715 (44.1)	(41.7–46.5)
30,000$ or over	907 (55.9)	(53.5–58.3)
Sexual orientation		
Homosexual or gay	1438 (82.8)	(81.0–84.6)
Bisexual	218 (12.6)	(11.0–14.1)
Other	81 (4.7)	(3.7–5.7)
Place of birth		
Canada	1082 (62.2)	(60.0–64.5)
Elsewhere	657 (37.8)	(35.5–40.1)
Tested for HIV in the previous 12 months		
No	729 (47.9)	(45.4–50.4)
Yes	793 (52.1)	(49.6–54.6)
Ever taken PEP in the past		
No	1535 (89.0)	(87.6–90.5)
Yes	189 (11.0)	(9.5–12.4)
HIV status of primary partner		
Not in a relationship	960 (65.1)	(62.7–67.6)
HIV-negative	366 (24.8)	(22.6–27.0)
HIV-positive	44 (3.0)	(2.1–3.9)
Unknown	104 (7.1)	(5.7–8.4)
Proportion of sexual encounters that happened in saunas		
None or less than half the time	1344 (83.5)	(81.7–85.3)
Half the time or more	266 (16.5)	(14.7–18.3)
Intentional condomless anal sex		
No	1330 (82.8)	(80.9–84.6)
Yes	277 (17.2)	(15.4–19.1)
HIV test result (reactive)		
No	1703 (98.0)	(97.4–98.7)
Yes	34 (2.0)	(1.3–2.6)
Previously had an STI		
No	1072 (61.7)	(59.4–64.0)
Yes	666 (38.3)	(36.0–40.6)

As shown in Table 2, just under 41 % of participants reported anal sex in the past 3 months with partners of unknown HIV status, about 3 % with HIV-positive partners whose viral load was detectable or unknown, and about the same proportion with HIV-positive partners whose viral load was undetectable. Consistent condom use, especially important with partners whose HIV viral load is detectable or unknown, ranged from 47 to 64 % depending on positioning (top or bottom) and type of sexual partner.

**Table 2:** Prevalence of items used in the latent class analysis (N = 1740)

	N (%)	(95 % CI)
Partners of unknown HIV status		
Anal sex as a top (yes)	711 (40.9)	(38.6–43.2)
Condom never used	85 (11.9)	(9.6–14.3)
Inconsistent condom use	228 (32.1)	(28.6–35.5)
Systematic condom use	398 (56.0)	(52.3–59.6)
Anal sex as a bottom (yes)	628 (36.2)	(33.9–38.4)
Condom never used	72 (11.5)	(9.0–14.0)
Inconsistent condom use	189 (30.1)	(26.5–33.7)
Systematic condom use	367 (58.4)	(54.6–62.3)
HIV-positive partners with unknown or detectable viral load		
Anal sex as a top (yes)	59 (3.4)	(2.5–4.2)
Condom never used	16 (27.1)	(15.4–38.8)
Inconsistent condom use	5 (8.5)	(1.2–16.8)
Systematic condom use	38 (64.4)	(51.8–77.0)
Anal sex as a bottom (yes)	48 (2.8)	(2.0–3.5)
Condom never used	14 (29.2)	(15.8–42.5)
Inconsistent condom use	4 (8.3)	(0.2–16.4)
Systematic condom use	30 (62.5)	(48.3–76.7)
HIV-positive partners with undetectable viral load		
Anal sex as a top (yes)	58 (3.3)	(2.5–4.2)
Condom never used	17 (29.3)	(17.2–41.4)
Inconsistent condom use	14 (24.1)	(12.8–35.5)
Systematic condom use	27 (46.6)	(33.3–59.8)
Anal sex as a bottom (yes)	54 (3.1)	(3.2–3.9)
Condom never used	16 (29.6)	(17.0–42.2)
Inconsistent condom use	8 (14.8)	(5.0–24.6)
Systematic condom use	30 (55.6)	(41.9–69.2)

### Identification of Latent Classes and Participants’ Sociosexual Characteristics According to Class

Table 3 presents estimated probabilities (EP) for each of the covariates within each latent class based on likely class membership. Table 4 presents multivariate analysis of the differences between classes based on sociosexual and health profile variables using a post hoc pairwise comparison [76]. For the sake of parsimony, we briefly describe each class using just some of the results presented in the tables, focusing on those that allow the classes to be distinguished from one another.

**Table 3:** Estimated probabilities (EP) of reporting each item and prevalence by class based on latent class analysis for a 5-class solution

Class Size	Class 1	Class 2	Class 3	Class 4	Class 5
	0.539 (N = 938)	0.218 (N = 380)	0.184 (N = 320)	0.031 (N = 54)	0.028 (N = 49)
Partners of unknown HIV status					
Anal sex (complete penetration) as a top					
No	0.861	0.208	0.253	0.633	0.534
Yes	0.139	0.792	0.747	0.367	0.466
Condom never used	0.084	0.208	0.006	0.253	0.224
Inconsistent condom use	0.169	0.588	0.069	0.243	0.360
Systematic condom use	0.747	0.204	0.925	0.504	0.416
Anal dipping (partial penetration) as a top					
No	0.997	0.476	0.641	0.776	0.691
Yes	0.003	0.524	0.359	0.224	0.309
Condom never used	0.968	0.463	0.141	0.166	0.402
Inconsistent condom use	0.000	0.493	0.114	0.583	0.334
Systematic condom use	0.032	0.044	0.745	0.251	0.264
Anal sex as a bottom					
No	0.914	0.320	0.219	0.645	0.547
Yes	0.086	0.680	0.781	0.355	0.453
Condom never used	0.139	0.213	0.000	0.158	0.137
Inconsistent condom use	0.231	0.610	0.000	0.312	0.329
Systematic condom use	0.630	0.177	1.000	0.530	0.534
Anal dipping as a bottom					
No	0.993	0.583	0.591	0.850	0.793
Yes	0.007	0.417	0.409	0.150	0.207
Condom never used	0.333	0.448	0.094	0.001	0.299
Inconsistent condom use	0.000	0.531	0.048	0.625	0.599
Systematic condom use	0.667	0.021	0.858	0.374	0.102
HIV-positive partners with unknown or detectable viral load					
Anal sex as a top					
No	0.991	0.986	0.989	1.000	0.149
Yes	0.009	0.014	0.011	0.000	0.851
Condom never used	0.323	0.352	0.000	0.000	0.274
Inconsistent condom use	0.000	0.000	0.277	0.000	0.096
Systematic condom use	0.677	0.648	0.723	1.000	0.630
Anal dipping as a top					
No	1.000	1.000	0.997	0.981	0.529
Yes	0.000	0.000	0.003	0.019	0.471
Condom never used	0.000	0.000	0.000	0.000	0.480
Inconsistent condom use	0.000	0.000	0.000	1.000	0.087
Systematic condom use	0.000	0.000	1.000	0.000	0.433
Anal sex as a bottom					
No	0.998	0.987	0.993	0.981	0.229
Yes	0.002	0.013	0.007	0.019	0.771
Condom never used	0.000	0.170	0.000	0.000	0.350
Inconsistent condom use	0.435	0.000	0.471	0.995	0.027
Systematic condom use	0.565	0.830	0.529	0.005	0.623
Anal dipping as a bottom					
No	1.000	1.000	0.997	0.981	0.548
Yes	0.000	0.000	0.003	0.019	0.452
Condom never used	0.000	0.000	0.000	0.000	0.545
Inconsistent condom use	0.000	0.000	0.000	1.000	0.045
Systematic condom use	0.000	0.000	1.000	0.000	0.410
HIV-positive partners with undetectable viral load					
Anal sex as a top					
No	0.995	0.989	0.993	0.222	0.897
Yes	0.005	0.011	0.007	0.778	0.103
Condom never used	0.000	0.232	0.000	0.381	0.000
Inconsistent condom use	0.551	0.232	0.000	0.225	0.200
Systematic condom use	0.449	0.536	1.000	0.394	0.800
Anal dipping as a top					
No	1.000	0.997	1.000	0.389	0.897
Yes	0.000	0.003	0.000	0.611	0.103
Condom never used	0.000	0.000	0.000	0.485	0.000
Inconsistent condom use	0.000	0.000	0.000	0.273	0.200
Systematic condom use	0.000	1.000	0.000	0.242	0.800
Anal sex as a bottom					
No	0.995	0.992	0.986	0.281	0.938
Yes	0.005	0.008	0.014	0.719	0.062
Condom never used	0.370	0.000	0.000	0.371	0.000
Inconsistent condom use	0.000	0.000	0.000	0.181	0.332
Systematic condom use	0.630	1.000	1.000	0.448	0.668
Anal dipping as a bottom					
No	1.000	0.995	1.000	0.500	0.938
Yes	0.000	0.005	0.000	0.450	0.062
Condom never used	0.000	0.000	0.000	0.482	0.000
Inconsistent condom use	0.000	0.500	0.000	0.148	0.333
Systematic condom use	0.000	0.500	0.000	0.370	0.667

**Table 4:** Differences between classes based on sexual behavior (multivariate analysis)

Variable	Class 1	Class 2	Class 3	Class 4	Class 5	Wald statistics	*P* value (for difference)
Tested for HIV in the previous 12 months^1^ (%)	47.4	55.2	56.2	72.7	67.8	9.9	0.042
Ever taken PEP in the past (%)	8.8^a^	12.8	11.3	28.7^a^	17.5	9.6	0.047
HIV status of primary partner (%)							
Not in a relationship	62.3	66.8	71.9	63.2	63.5	115.7	<0.0001
HIV-negative	31.5^a,b^	15.4^a^	19.2^b^	17.3	14.0		
HIV-positive	2.7^a,b^	1.4^c,d^	1.1^e,f^	17.3^a,c,e^	19.1^b,d,f^		
Unknown	3.5^a^	16.4^a,b^	7.8^b^	2.2	3.5		
Proportion of sexual encounters that happened in saunas (half the time or more) (%)	12.6^a^	21.6	20.9^a^	15.0	25.5	22.9	0.0001
Intentional condomless anal sex (%)	10.8^a,b,c^	46.9^a,d,e^	0.0^b,d,f,g^	35.3^c,f^	17.3^e,g^	466.1	<0.0001
Reactive HIV test result(%)	1.3	3.4	2.0	3.9	2.1	7.2	0.130
Previously had an STI (%)	31.0^a,b,c^	50.8^a,d^	37.9^d^	64.2^b^	59.0^c^	41.5	<0.0001
Attitudes towards HIV testing^1^ (mean)^2^	6.67	6.67	6.69	6.72	6.63	11.1	0.026

Letters in superscript (a, b, c, d, e, f, g): proportions and means with the same superscript letter statistically differ at *P* < 0.005 in post hoc pairwise comparisons (adjusted value for a familywise Type I error rate set at 0.05)

^1^Post hoc tests did not reveal any significant pairwise mean differences when adjusting the critical value of *p* to reflect the number of tests we performed

^2^ *Would rather know your HIV status to make the right decisions for your own health and the health of others?* Scale varying from 1 “Strongly disagree” to 7 “Strongly agree”

#### Class 1

The most prevalent pattern (class 1) includes just over half the sample (53.9 %) (Table 3). These participants used a strict form of serosorting as their main strategy in that they generally avoided anal sex with partners of unknown HIV status (EP: top, over 0.86; bottom, over 0.91) and HIV-positive partners, regardless of viral load (EP: top, over 0.99; bottom, over 0.99). When having anal sex with partners of unknown status, a high proportion used condoms consistently (EP: top, 0.75; bottom, 0.63). This concords with their sociosexual profile (Table 4) in that they were more likely to be in a seroconcordant HIV-negative couple than any other class (31.5 %), less likely to have met partners in a sauna in the past 3 months (12.6 %), and less likely to report having previously had an STI (31.0 %).

#### Class 2

The second most common pattern (class 2) describes 21.8 % of participants (Table 3). These participants also used a fairly strict form of serosorting in systematically avoiding anal sex with HIV-positive partners, regardless of viral load (EP: top, over 0.98; bottom, over 0.98). They were more likely to have had anal sex with partners of unknown status than any other class, but more often as a top than as a bottom (EP: top, 0.79; bottom, 0.68) which suggests that strategic positioning may have been used with these partners. A minority reported systematic condom use during anal sex (EP: top, 0.20; bottom, 0.18). As shown in Table 4, the proportion who reported intentional condomless anal sex in the past 3 months was higher than in any other class (46.9 %) and participants in class 2 were among those most likely to report having previously had an STI (50.8 %).

#### Class 3

Nearly 1 in 5 participants (18.4 %) (Table 3) fit into the third most prevalent pattern (class 3). Participants in this class stand out in being more likely to report anal sex with partners of unknown HIV status (EP: top, 0.75; bottom, 0.78) but tended to use condoms systematically with this type of partner (EP: top, 0.93; bottom, 1.00). This strategy was combined with avoidance of anal sex with HIV-positive partners, regardless of viral load (EP: top, 0.99; bottom, 0.99) (strict serosorting). This is consistent with their sociosexual profile (Table 4) in that they were among the classes most likely to report having met their partners in a sauna (20.9 %). They were the least likely to report intentional condomless anal sex in the past 3 months (0.0 %), and among the least likely to report having previously had an STI (37.9 %).

#### Class 4

The last two patterns (classes 4 and 5) are both characterized by anal sex with HIV-positive partners and include a small proportion of participants. About 3 % (3.1 %) of the sample falls into class 4 (Table 3). These participants make use of diverse non-condom based strategies including consideration of viral load. They were more likely to report anal sex with HIV-positive sexual partners whose viral load was undetectable (EP: top, 0.78; bottom, 0.72) but more often as a top than as a bottom, suggesting the use of strategic positioning [6]. Overall, they did not use condoms systematically (EP: top, 0.39; bottom, 0.45) but condom use was higher if they were the bottom with these partners. They were the least likely to report anal sex with HIV-positive partners whose viral load was detectable or unknown (EP: top, 0.00; bottom, 0.02) and a high proportion did not have sexual partners of unknown HIV status (EP: top, 0.63; bottom, 0.65). As shown in Table 4, they were the most likely to have been tested for HIV in the past year (72.7 %) and to say they were checking their HIV status in order to make better health decisions for themselves and their partners, suggesting a favorable attitude toward testing. They were also among the most likely to be in a relationship with an HIV-positive partner (17.3 %). They were the most likely to have used PEP in the past (28.7 %), among the most likely to have had intentional condomless anal sex in the past 3 months (35.3 %), and the most likely to have previously had an STI (64.2 %).

#### Class 5

Class 5 includes 2.8 % of the sample (Table 3). These participants were among the least likely to report anal sex with partners of unknown HIV status (EP: top, 0.47; bottom, 0.45) and with HIV positive partners whose viral load was undetectable (EP: 0.10; bottom, 0.06). However, they reported more anal sex with HIV-positive partners whose viral load was unknown or detectable than any other class (EP: top: 0.85, bottom, 0.77). Condom use with partners of unknown HIV status was reported by roughly half (EP: top, 0.42; bottom, 0.53), but nearly two-thirds reported systematic condom use with HIV-positive partners whose viral load was unknown or detectable (EP: top, 0.63; bottom, 0.62). As shown in Table 4, these participants were the most likely to report being in a relationship with an HIV-positive partner (19.1 %), among the most likely to have used PEP in the past (17.5 %), and among the most likely to have previously had an STI (59.0 %). Nearly 68 % said they had been tested for HIV in the past 12 months.

## Discussion

Among MSM accessing rapid HIV testing at a community testing site, this study identified five patterns with respect to how condoms are used with HIV-positive partners or partners of unknown HIV status in conjunction with non condom-based behavioral and biomedical risk reduction strategies. As summarized in Table 5, strict serosorting is a key strategy for classes 1, 2 and 3. Participants in class 1 strictly avoid serodiscordant partners and partners of unknown HIV status. Those in classes 2 and 3 are sexually active with partners of unknown status but practice strict serosorting by avoiding anal sex with HIV-positive partners, regardless of viral load. Participants in class 2 also use strategic positioning whereas participants in class 3 use condoms consistently with partners of unknown status (condom serosorting [6], especially when these participants are the bottom (condom positioning) [6]. Participants in classes 4 and 5 stand out by virtue of the relations they have with serodiscordant partners, in the case of class 4 with partners whose viral load is undetectable (akin to consideration of viral load or viral load serosorting) [6] and in the case of class 5, with partners whose viral load is detectable or unknown. The data point to a probability that strategic positioning (more often being the top than the bottom) is being used in both classes along with condom positioning (more frequent condom use if bottoming). These two classes contain the highest proportions of participants who said they had been tested for HIV in the past twelve months and had ever used PEP.

**Table 5:** Summary of patterns in the use of sexual risk reduction strategies by class

Strategies	Class 1	Class 2	Class 3	Class 4	Class 5
Based on behavior in the past three months					
Strict serosorting for HIV+ and HIV? (unknown status) partners	*✓✓*				
Strict serosorting for HIV+ partners		✓✓	✓✓		
Condom use with HIV? partners (condom serosorting)	✓		✓✓		
Strategic positioning with HIV? partners		✓			
Strategic positioning with HIV+ partners, undetectable viral load				✓	
Strategic positioning with HIV+ partners, detectable or unknown viral load					✓
Taking viral load into consideration				✓✓	
Condom use when bottom with HIV? partners (condom positioning)			✓		✓
Condom use when bottom with HIV+ partners, undetectable viral load				✓	
Condom use with HIV+ partners, detectable or unknown viral load (condom serosorting)					✓
Based on past behaviors					
Tested for HIV in the previous 12 months	✓		✓	✓✓	✓✓
Ever taken PEP in the past				✓✓	✓

HIV? (unknown status); HIV+ (seropositive status); ✓✓ primary strategy; ✓ secondary strategy

All participants use a range of risk reduction strategies. This is significantly higher than what has been found in other studies [5–8, 42, 48]. Selection bias likely explains this difference since recruitment took place at Spot, a community-based HIV testing site. Participants are, for the most part, still taking precautions and those in class 1 and 3 (72 %) are using combinations that provide protection. Each of the other classes (2, 4 and 5) have their particular degree of risk. With the exception of class 3, consistent condom use is not the most prevalent strategy. Across the themes explored in the data and inspired by the approach taken by Prestage et al. [9], specific profiles can be discerned: the uncertainty of encounters with partners of unknown HIV status appears to motivate participants in class 3 to opt for more certainty by using condoms; participants in class 1 appear to feel that “nothing is safe”, avoiding anal sex with HIV-positive partners and partners of unknown status; finally, participants in class 4 share the belief, observed in studies on serodifferent relationships [9, 49, 81], that risk can be reduced without using condoms and opt primarily for strategic positioning and consideration of viral load.

Serosorting is practiced in various forms by 94 % of participants (classes 1, 2 and 3), a much higher proportion than in other studies [7, 8, 37, 45, 49, 50]. Researchers have observed that serosorting among HIV-negative men is associated with having recently had an HIV or STI test, condomless anal sex with a partner of different or unknown HIV status, having had an STI in the past 12 months, having had 10 or more partners, and using the Internet to meet sexual partners [8, 37, 82]. This profile is similar to Class 2, the group most at risk comprising 1 in 5 participants (22 %). It differs markedly from the profiles of classes 1 and 3 (72 %), the largest group whose use of serosorting combined with testing offers a measure of protection. For these lower risk participants, regular testing seems to provide reassurance and serve as a way to confirm the effectiveness of their risk reduction choices. In contrast, the use of strict serosorting limited only to HIV-positive partners among participants in class 2 does not provide risk reduction given that these participants have anal sex, most of the time without a condom, with partners of unknown status. Those who assume that their sexual partners are HIV-negative will need to get tested frequently since the effectiveness of this strategy depends on accurate knowledge of HIV status (one’s own and one’s partners) and on the proportion of HIV-positive individuals in the community who are unaware they are infected [44, 81], estimated to be 14 % among MSM in Montreal [4].

The prevalence of strategic positioning (28 %) is at the upper limits of what has been reported in other work [5, 6, 8, 32, 48]. Participants in classes 4 and 5 were more likely to report being a top rather than a bottom when having anal sex with HIV-positive partners and those in class 2, with partners of unknown HIV status. However, it is not possible to determine if this reflects sexual preference or deliberate and strategic positioning [8, 48, 83] since our data group together all strategies used over the past 3 months without specifying which strategies were used for each specific sexual encounter. In classes 4 and 5, strategic positioning seems to be combined with more systematic testing. In several recent studies, the proportion of MSM who had been tested in the last 12 months was around 58 % [32, 61, 62], close to the proportions observed in classed 1, 2, and 3 but significantly lower than classes 4 and 5. For these participants, in particular class 4, testing is primarily motivated by the importance of knowing their HIV status in order to make better health decisions.

The prevalence of PEP use across all classes is higher than in other research [32, 68, 69, 71, 72], ranging from 9 % in class 1–18 % and 29 % in Classes 5 and 4 respectively. PEP promotion campaigns undertaken in Montreal’s gay community in recent years may explain this higher prevalence. In Class 4, consideration of a partner’s viral load is also used. A number of studies have shown an association between undetectable viral load and condomless anal sex among HIV-negative men in serodifferent relationships [47, 84, 85].

### Limitations

This study is based on a convenience sample. About 6 % of those invited did not want to participate in the study and no data was collected to describe them. Participants went to Spot because they wanted to check their HIV status, meaning they had some awareness of risk and degree of interest in reducing their risk. Since all participants were residents of Quebec, care must be taken in generalizing to other MSM populations based on these findings. To minimize inaccuracies in self-reported information arising from misunderstood questions, questionnaires were administered during a face-to-face appointment. To counter social desirability issues with respect to sexual behavior, pre- and post-test counseling was provided by community-based peers. Data was gathered on sexual practices in the previous 3 months to reduce the potential for bias from not remembering properly, but we cannot say with certainty that participants consistently used the risk reduction strategies identified in this study and we have not explored the full range of possible combinations since some strategies, such as withdrawal before ejaculation, were not examined.

Moreover, the latent class analysis we have undertaken does not take into account the relationship status (regular, casual, one-night) of sexual partners in the past 3 months or whether a negotiated safety agreement was used. Given that factors such as familiarity and trust can influence decision-making relative to risk reduction strategies [86], information about the relationship status for each sexual partner would have provided additional insight to guide our interpretation of the data with respect to how participants made these decisions. However, we did take relationship status into consideration in the second part of the analysis when describing the profiles that characterize each class (Table 4). This analysis shows that participants in class 1 are more likely than those in other classes to be in a relationship with an HIV-negative partner, whereas participants in classes 4 and 5 are more likely to be in a relationship with a serodiscordant partner. These distinctions suggest that relationship status plays a role in shaping patterns of sexual behavior and the use of risk reduction strategies among participants in this study.

We have extrapolated the strategies used by participants based on an analysis of their behavior in the past 3 months rather than by analyzing answers to questions about which strategies they use in a given context. It is therefore difficult to argue that the five patterns that have been identified represent explicitly intentional, deliberate strategies [48, 83]. The relevance of a behavioral focus is that it allows for a more accurate assessment of the risks that people are actually taking.

### Implications for Research and Intervention

Similar analyses should be undertaken in other populations and in particular among HIV-positive MSM to establish whether these patterns can be observed elsewhere and if other profiles can be identified. In addition to exploring the full set of risk reduction strategies an individual may use, the specific strategies used for each sexual encounter also need to be studied to better understand context-dependent factors that influence which risk reduction strategies are combined together [8]. Particular attention will need to be paid to understanding the extent to which PrEP is used in combination with condoms or as a replacement for condom use. To improve how interventions are tailored, a more detailed inventory of psychosocial and sociocultural determinants that characterize each of the profiles should be developed. As well, longitudinal studies are required to monitor emerging strategies such as negotiating condomless anal sex based on PrEP use and trends in risk compensation and the use of seroadaptive practices associated with the use of new prevention technologies [49, 87, 88].

In the Montreal context, awareness of the limits of serosorting and the conditions under which it can be effective must be increased since this strategy is used by the vast majority of participants (94 %) but in a potentially ineffective way by many (class 2, 22 %). With respect to the strict type of serosorting used by participants in classes, 1, 2 and 3, this strategy often involves discrimination against HIV-positive individuals. Particular emphasis should be placed on disseminating accurate information about infectiousness as it relates to viral load and the higher risk associated with a sexual partner of unknown HIV status compared to an HIV-positive partner with an undetectable viral load.

At the individual level, combination prevention for MSM will involve the adoption of personalized risk reduction strategies used before, during, or after sex and tailored to a person’s HIV status, needs, and sexual preferences. The high prevalence of self-reported STIs in the past and the low prevalence of condom use in this sample reinforce the importance of community-wide condom promotion and distribution. Interventions are also required to help MSM develop seroadaptive skills, adopt testing routines based on sexual lifestyle, consolidate self-efficacy with respect to the disclosure and discussion of HIV status, and assess the risk inherent to each sexual encounter [6].

## Conclusion

Participants in this study make strategic use of condoms to some extent but also use other risk reduction strategies shaped by a range of lifestyles. The diversity of strategies suggests that MSM are integrating health messages to a certain degree and continue to adapt their sexual practices in light of available scientific and medical evidence. To reduce HIV transmission, combination HIV prevention is needed at both community and individual levels to facilitate access to a range of prevention options and their integration into different sexual activities. Alongside seroadaptive practices, condoms continue to serve as an effective behavioral strategy for many MSM. Meanwhile, the effectiveness of biomedical strategies such as PrEP and PEP largely depends on behavioral dimensions such as adherence to treatment [89–91]. These are among the reasons why it is crucial that behavioral strategies be strengthened as part of this combined approach.
